# Mining the human proteome for conserved mechanisms

**DOI:** 10.1186/1471-2105-16-S3-A6

**Published:** 2015-02-13

**Authors:** Stefan Naulaerts, Pieter Meysman, Wim Vanden Berghe, Kris Laukens

**Affiliations:** 1 ADReM research group, Department of Mathematics and Computer Science, University of Antwerp, Belgium; 2 Biomedical Informatics Research Center Antwerp (biomina), University of Antwerp/Antwerp University Hospital, Belgium; 3 Laboratory of Protein Science, Proteomics and Epigenetic Signaling (PPES), Department of Biomedical Sciences, University of Antwerp, Belgium

## Background

All cells are subject to ever-changing environments to which they have to adapt, using their sensory system to provide input for the regulatory systems that integrate the information and trigger the eventual effectors. These cascades constitute a very complex cellular wiring that is highly relevant due to its medical importance. The omni-present application of high-throughput analysis techniques has resulted in an unprecedented level of available detail about gene expression and various aspects of cellular proteins, such as abundance, function and localization, often captured in well-curated compendia that are publicly available.

Although these information-rich inventories exist, the adaptive nature of protein complexes and signalling cascades remain poorly understood, as the current predominant approaches are not always suited to describe the associations between proteins. For example, binary protein interactions do not necessarily occur in vivo as the proteins could be expressed in different compartments of the cell or at different time points. This severely complicates the analysis of any protein interaction data. It thus remains a challenge to find out how biological entities cooperate to regulate cellular response to stimuli.

## Methods

We used an integrative method, reliant on advanced pattern mining approaches to gain a deeper understanding of protein network dynamics. To this end, we created a compendium consisting of a large amount of proteomics papers for *Homo sapiens *that report differentially expressed proteins in cell lines. Next, we analysed this collection with frequent itemset mining to identify proteins that are often co-occurring in publications and used these patterns as the backbone structure of our further analysis. These patterns of co-occurring proteins were enriched with additional attributes, such as gene expression correlation, protein localization and functional coherence metrics derived from the Gene Ontology tree [1] and used as a filter on top of an integrated binary protein interaction network, obtained by fusing several of the most popular resources.

## Results

We found that several proteins and GO-functions, such as transcriptional regulation, are consistently reported and deemed significant regardless of the research topic. Furthermore, we were able to find associations across the various "omics" levels that are conserved in a wide range of human cancers and managed to identify lists of frequently occuring patterns that can be used to classify between pre- and post-metastasic tumour development.

## Conclusions

Pattern-based analysis on multiple "omics" levels can be used to identify the cellular logic circuits and holds many promising applications in the biotechnological and biomedical areas.

## References

[B1] Ashburner M Ball C Blake J Botstein D Butler H Cherry J Davis A Dolinski K Dwight S Eppig J Gene ontology: tool for the unification of biology Nat Genet 2000 25 1 25 29 10.1038/75556 10802651 PMC3037419

